# Doppler sonar analysis of swallowing sounds in normal pediatric individuals

**DOI:** 10.1016/S1808-8694(15)30522-X

**Published:** 2015-10-18

**Authors:** Cibele Fontoura Cagliari, Ari Leon Jurkiewicz, Rosane Sampaio Santos, Jair Mendes Marques

**Affiliations:** 1 Communication Disorders, Head of the Speech and Audiology Department - Pequeno Príncipe hospital; 2 Anatomy - UNIFESP, Adjunct Professor - Graduate Program in Communications Disorders - Tuiuti University - Paraná; 3 Communications Disorders - Tuiuti University - Paraná, Speech and Audiology Department Professor Tuiuti University - Paraná; 4 Geodesic Sciences - Federal University of Paraná - Professor of the Graduate Program in Communications Disorders Tuiuti University - Paraná. Tuiuti University - Paraná

**Keywords:** swallowing, doppler effect, pharynx, larynx

## Abstract

Among the methods for assessing swallowing sounds - videofluoroscopy modified barium study, fiberoptic swallowing endoscopy, neck auscultation through a microphone, accelerometer and, more recently, the Doppler sonar - we have chosen the latter.

**Aim:**

to analyze swallowing sounds by cervical auscultation using Doppler sonar, in a population between 2 and 15 years without oro-pharyngeal dysfunction.

**Study design:**

cross-sectional historical cohort.

**Materials and methods:**

we investigated 90 individuals in Curitiba (2006/2007). The population was separated by age into 3 groups: from 2 to 5 years, from 5 to 10 years of age and from 10 to 15 years of age. We obtained the average values for frequency, intensity and swallowing duration for saliva, liquid and pasty foods).

**Results:**

objective and measurable data were obtained. Significance related to gender was found in certain age groups and consistencies, under all the studied variables, except swallowing time.

**Conclusion:**

neck auscultation using Doppler sonar is a sensitive method to detect swallowing sounds. There was swallowing interference associated with saliva and the other tested food types and with the biological development of the age range being studied. It is an easy to apply method, not expensive and non-invasive.

## INTRODUCTION

An instrumental assessment together with the clinical exam of swallowing ultimately contributes to the definition of sequential approaches. Mechanisms which make this practice feasible require new methodologies to be created, tested and explored.

Neck auscultation by Doppler sonar is an innovative technique, characterized as a non-invasive method, without radiation exposure, easy to apply and of low cost^1^, enjoying much credibility gain in the clinical assessment of swallowing^2^.

All muscle, bone, cartilage and mucosa structures movement associated with food passage produces distinct and successive sounds^3^. Neck auscultation is defined as a method used to hear swallowing sounds with an amplifier during the pharyngeal phase^4^.

This method involves the placement of a sensor in the individual's neck in order to hear and/or record the acoustic signals captured by a microphone^5^ in order to mainly assess pharyngeal swallowing competence and its interaction with breathing. Food transit sounds can be assessed, just like the mild larynx and pharynx movements which precede and succeed food passage^6^.

There is a lot of competitive noise in neck auscultation because the neck is a relatively small region, with constant and significant acoustic activity. Most of the noise is hydraulic in nature because of blood vessels, secretions, CSF and air passing through^7^.

Prior studies on neck auscultation included acoustic analyses^2^, physiologic measures,^8^ processing and instrumental matters^9^.

One of the broadly investigated aspects associated with methodology is the place for pharyngeal swallowing sound detection, where there is a better signal-to-noise ratio. Using an accelerometer, we scanned 24 neck sites. Of these, three were referred to use during neck auscultation, and the area on the lateral trachea, immediately below the cricoid was selected as the best site for swallowing sound detection^10^.

The most prominent acoustic characteristic of the swallowing sound corresponding to food movement through the upper esophageal sphincter is found where the movements of the hyoid bone, the larynx and the epiglottis contributes to the swallowing acoustic signal^11^.

The respiratory action of the upper airways and the sudden changes in the respective muscles during the swallowing pharyngeal phase are also described as sound components^12^.

Changes in spectral characteristics are caused by an increase in food passing velocity, corresponding to the onset of pressurized flow to the stomach^8^.

Three swallowing sound components are considered: the first is a weak signal associated with larynx elevation and the food passing through the pharynx; the second, and stronger sound, is associated with the opening of the upper esophageal sphincter; and the third, a weak sound, is associated to the laryngeal downwards movement after swallowing^13^.

The swallowing acoustic landmark can be discussed in terms of acoustic signal frequency and duration, and sound wave amplitude^14^. The analysis of such characteristics in the swallowing of liquid and solid consistencies must be carried out in relation to gender and age^15^.

The parameters which define swallowing and its disorders in adults can not be always used in children, because of differences in anatomical structure relations^16^. The pediatric larynx is not simply a miniature of the adult larynx, because there are significant differences in size, its location in relation to the spine, in the make up of cartilage and soft tissue and adaptation to the environment^17^, ^18^.

Sound is a mechanical wave which moves through mediums, produced by a vibration source. Sound-frequencies higher than 25,000Hz, not hearable by humans, are known as ultrasounds^19^. Ultrasonography is based on the phenomena of sound-tissue interaction, that is, based on the transmission of a sound wave through the medium one can see the mechanical properties of tissues. The transducer is the part of the equipment which is responsible for generating, transmitting and capturing ultrasound as it turns electric power into ultrasound power and vice-versa. One of the main peculiarities associated with ultrasound is the very possibility of doing a non-invasive study through the Doppler effect.

The Doppler effect is defined as an alteration in the frequency sensation resulting from a situation in which the sound source is mobile, moving at a constant velocity and the receiver is stationary in some point of the trajectory. As the sound source approaches the receiver, the latter receives a larger number of waves per unit of time (higher frequency) and as it moves away it receives a lower number of waves (lower frequency)^20^.

In continuous Doppler, the signal emitted can be represented by a continuous sinusoidal wave, which amplitude is associated to pressure variations in the propagation mean or to particle shift^21^. The computer is capable of digitalizing the sounds and processing the noise produced by swallowing in wave format visual representations.

The feasibility of using the continuous Doppler sonar as a tool to help assess swallowing sounds and to identify acoustic parameters was shown in 50 normal adults swallowing saliva, liquid and pasty food^1^.

## OBJECTIVE

Our goal with the present study was to analyze swallowing sounds in individuals from 2 to 15 years of age without oropharyngeal dysphagia obtained through neck auscultation with the Doppler sonar.

## MATERIALS AND METHODS

We studied 90 individuals classified according to Chart 1. In order to classify them by age range, months were written as fractions of a year.

**Chart 1:** Individual distribution in relation to age and gender.

Age Range	From 2 to 5 years		From 5 to 10 years		From 10 to 15 years	
Gender	M	F	M	F	M	F
n	15 (16,66%)	15 (16,66%)	15 (16,66%)	15 (16,66%)	15 (16,66%)	15 (16,66%)

M= male; F= female; n= number of individuals studied

Inclusion criteria were minimum age of 2 years and maximum age of 15 years and normal swallowing in all its phases, proven by a prior evaluation carried out by a speech therapist.

Exclusion criteria were respiratory difficulty of any origin and structural or functional alterations which could impact swallowing, information obtained from parents and guardians and the assessment of the researcher.

**Figure 2:**
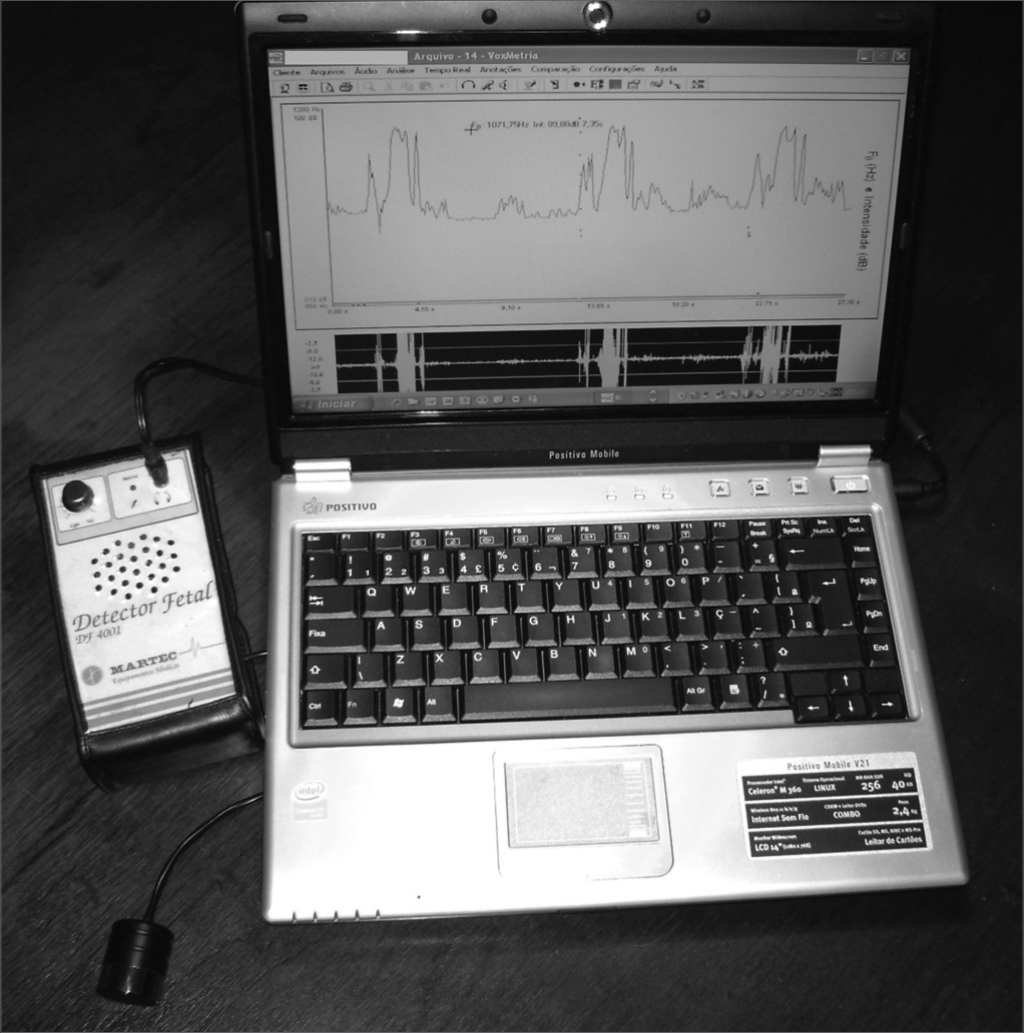
Doppler sonar coupled to a notebook computer

The continuous Doppler device was coupled to a Positivo notebook computer with an Intel Celeron M360 1.4GHz processor, 240MB of RAM memory, video card with integrated graphic accelerator Via UniChrome PRO IGP and 64MB of memory, Combo Drive (DVD player - CD recorder), integrated high definition ALC655 audio compatible with the AC'97, with Windows XP Professional operational system (Fig. 2).

**Figure 3:**
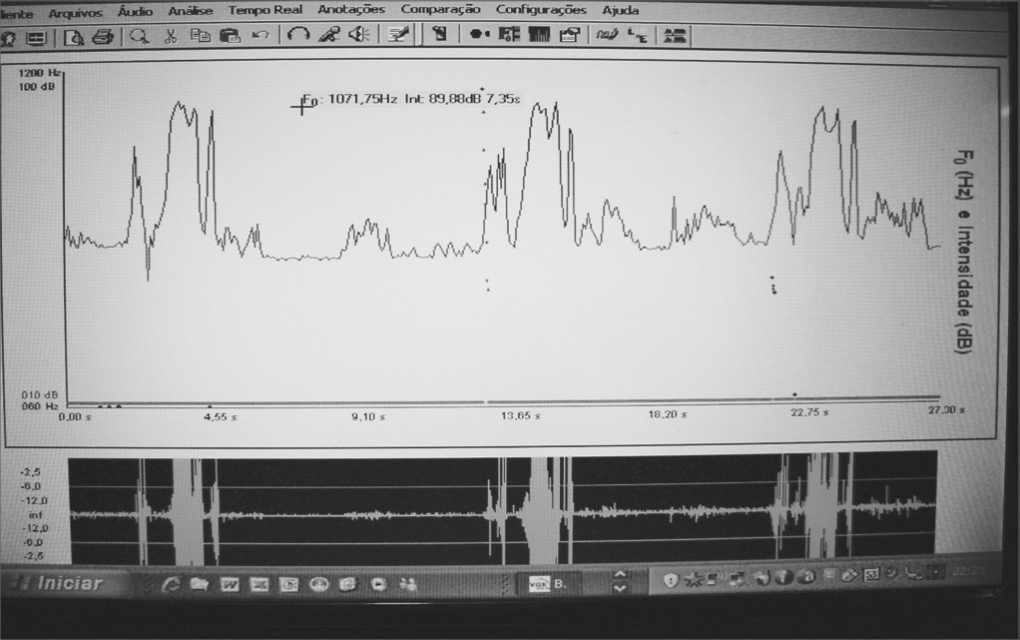
Voxmetria software tracing

The acoustic signals were recorded and analyzed by the VoxMetria software, version 2.8h22, through the voice analysis file (Fig. 3).

### Equipment and Software

**Figure 1:**
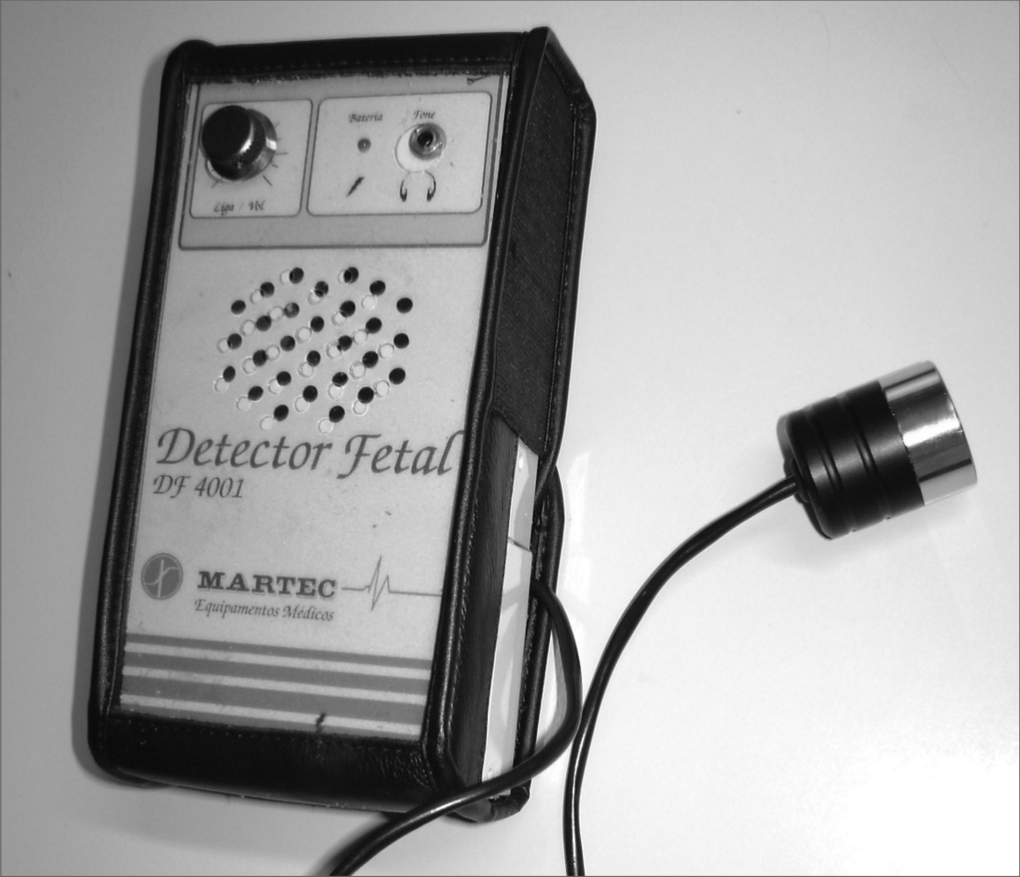
Sonar Doppler sonar

We used a portable ultrasound detector (DF-4001 model from Martec) with a flat disc transducer of a single crystal (Figure 1), providing the interface for the Doppler. The ultrasound frequency by the Doppler effect was 2.5MHz, with a 10mW/cm^2^ output. The sound output power was 1W.

### Standardizing Food Consistency

Liquid food consistency was defined as the swallowing of 2.5ml of water given in a disposable cup, and pasty food as the swallowing of 2.5ml of the Danoninho®[1] refrigerated yogurt-like product, given in a spoon.

### Procedures

In order to capture swallowing sounds, the individual was instructed to remain seated and have his neck free for access.

We made sure that everyone, specially the younger ones, were able to understand verbal commands which were given - under strict compliance with the protocol established. Participant collaboration was uniformly satisfactory.

Subjects were instructed to swallow the entire volume offered in one single gulp. Swallowing took place immediately after the investigator's command, about three seconds after placing the transducer on the right neck lateral region. A minimum of three swallowing samples were collected from each one.

Saliva swallowing sounds were the first captured, followed by those of liquid and then the pasty substance. Three minutes passed between the swallowing of each different substance.

[1] Danoninho® nutritional make up: skim milk, strawberry mix (water, fruit-oligosaccharides, fructose, sugar, strawberry, calcium, zinc, modified starch, vitamins A and D, carmin cochonilha natural dye, citric acid acidulant, carrageen thickener and xantan gum, carboxymethylcellulose and guar gum stabilizers, potassium sorbate preservative and aromatizer), sugar, milk cream, calcium chloride, lacteous ferment, clotting agent and potassium sorbate preservative).

### Swallowing sounds capture by the Doppler Sonar

The Doppler Sonar transducer was placed on the right lateral neck, on the tracheal lateral border, just below the cricoid cartilage.

The Doppler device volume was set to One, for proper audio signal capture by the VoxMetria software and lower external noise interference.

When the noise produced by the common carotid artery pulsation was captured, in some samples, the transducer was repositioned and a new sound capture ensued.

The ultrasound energy beam emitted by the transducer was guided to form a 30° to 60° angle. We used contact Carbogel gel to reduce ultrasound scattering and to increase its eco and body transmission.

### Acoustic analysis of the Swallowing Sound Signal

**Figure 4:**
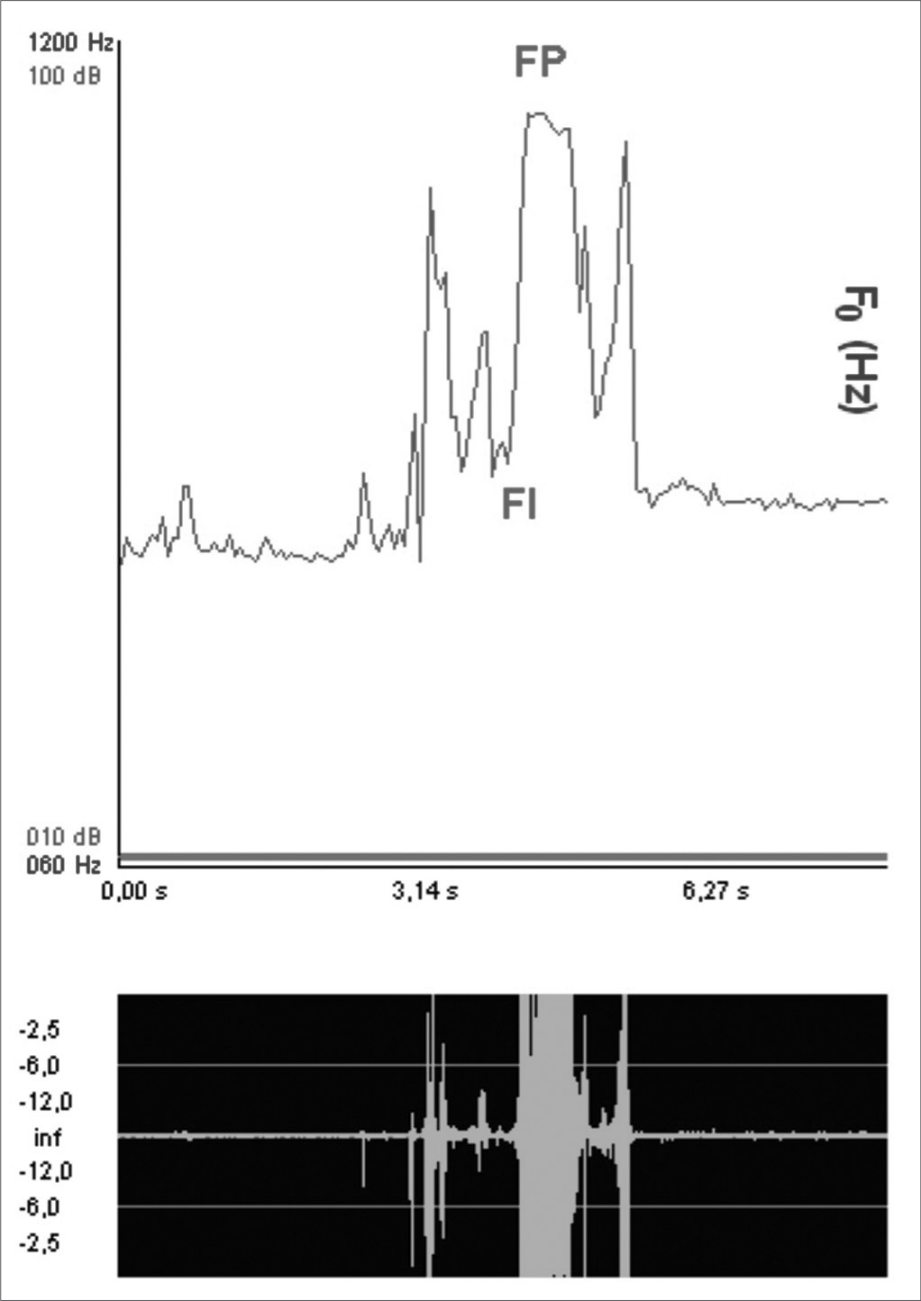
Sound wave peak and initial frequencies
c) Sound wave initial intensity (II): intensity at acoustic signal onset, with the window between 10 and 100dB;
d) Sound wave peak intensity (IP): intensity at the highest point of the acoustic signal shift, with the aforementioned intensity window (Fig. 5);

Five variables were measured for each saliva swallowing effort and for the liquid and pasty substances:

a) Sound wave initial frequency (FI): frequency at acoustic signal onset, with the frequency window between 60 and 12000Hz;
b) Sound wave peak frequency (FP): frequency at the point of highest acoustic signal shift, with the aforementioned frequency window (Fig. 4);

**Figure 5:**
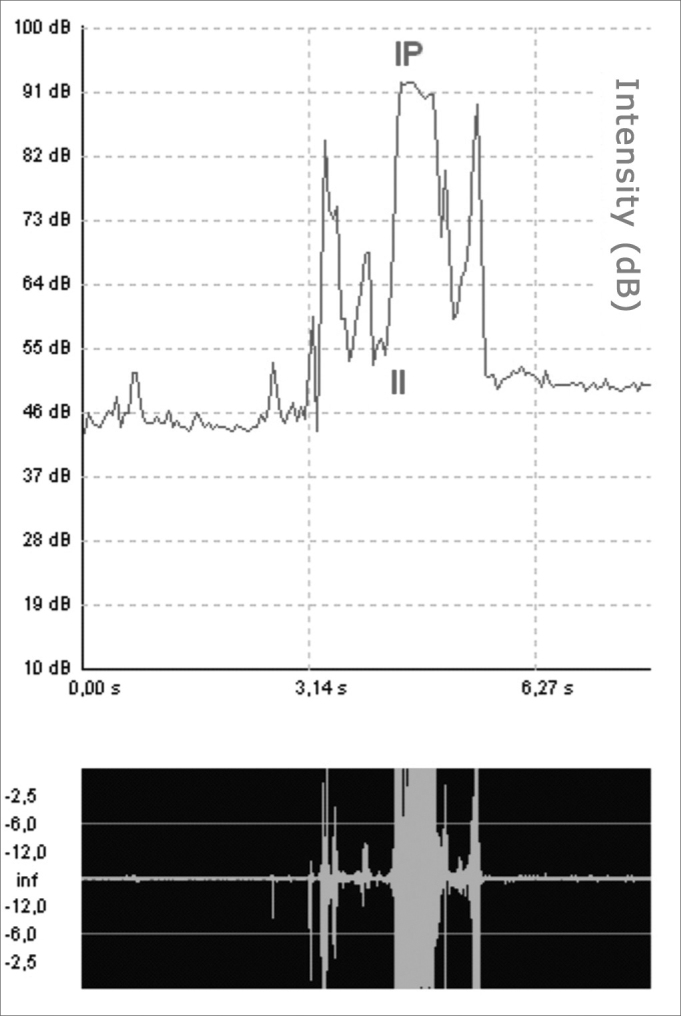
Sound wave peak and initial intensities
e) Swallowing time (T): time between the beginning and the end of the analyzed acoustic signal, measured by means of an audio signal, in seconds (Fig. 6).

**Figure 6:**
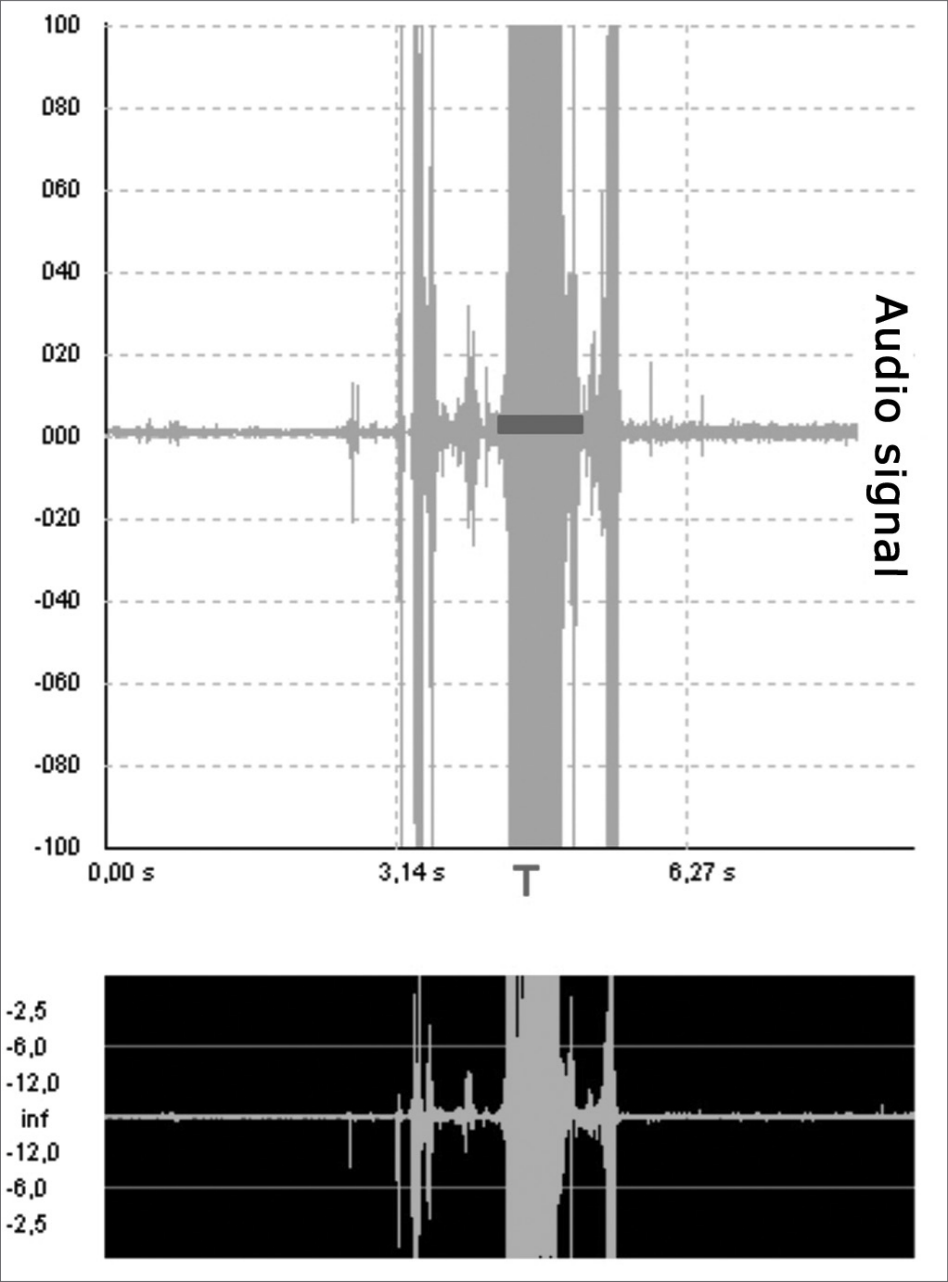
Swallowing time

In order to plot the swallowing sounds from the individuals in terms of frequency, intensity and swallowing duration, we selected the sample considered the best audio and visual presentation.

In order to specify saliva swallowing and that of liquid and pasty consistency foods, numbers and colors were standardized to the acronyms of the variables mentioned:

a) number 1 (FI1, FP1, II1, IP1, T1) and the green color for saliva swallowing;
b) number 2 (FI2, FP2, II2, IP2, T2) and the red color for liquid food;
c) number 3 (FI3, FP3, II3, IP3, T3) and the blue color for pasty food.

The statistical methodology was made up of descriptive (mean) and inferential (significance test) analysis approach. In order to analyze the significance of acoustic parameters between men and women at each age range for saliva, liquids and pasty foodstuff, the Student t test was used.

The ANOVA test was used in order to check the significance within one age range for the three consistencies, and in the same consistency for the three age ranges based on the profile found at each one of the five variables.

The significance level was equal to 0.05 where p<0.05 is considered significant. On the tables, significant values are stressed with an asterisk.

## RESULT

### Mean values and significance according to gender

Mean and significance values according to gender for saliva and liquid and pasty foodstuff for the different age ranges were checked in the initial and peak frequencies (Table 1, Table 2), initial and peak intensities (Table 3, Table 4) and swallowing times (Table 5).

**Table 1:** Mean values and results from the tests comparing initial frequencies according to gender.

CONSISTENCY	AGE RANGES (YEARS)	MALES	FEMALES	P
	2 to 5	721,91	676,16	0,0394 *
Saliva	5 to 10	707,18	705,28	0,9235
	10 to 15	707,42	770,12	0,0003 *
	2 to 5	710,96	704,57	0,7449
Liquid	5 to 10	746,85	703,62	0,0223 *
	10 to 15	707,66	751,60	0,0479 *
	2 to 5	715,18	716,92	0,8629
Paste	5 to 10	731,41	696,26	0,0428*
	10 to 15	700,77	746,85	0,0392*

**Table 2:** Mean values and results from the peak frequency comparison tests according to gender.

CONSISTENCY	AGE RANGES (YEARS)	MALES	FEMALES	P
	2 to 5	1078,63	1056,58	0,1472
Saliva	5 to 10	1094,55	1081,48	0,0628
	10 to 15	1092,41	1105,23	0,0434*
	2 to 5	1086,95	1093,36	0,4402
Liquid	5 to 10	1106,90	1100,01	0,0554
	10 to 15	1105,00	1107,37	0,2124
	2 to 5	1074,36	1073,41	0,9302
Paste	5 to 10	1096,68	1075,55	0,0277 *
	10 to 15	1091,94	1103,85	0,0636

**Table 3:** Mean values and results from the initial intensity comparison tests according to gender.

CONSISTENCY	AGE RANGES (YEARS)	MALES	FEMALES	P
	2 to 5	62,08	58,22	0,0394 *
Saliva	5 to 10	61,09	60,94	0,9229
	10 to 15	61,11	66,06	0,0003 *
	2 to 5	61,45	60,88	0,7444
Liquid	5 to 10	64,22	60,81	0,0223*
	10 to 15	61,13	64,60	0,0479*
	2 to 5	61,58	61,86	0,8631
Paste	5 to 10	63,00	60,23	0,0428 *
	10 to 15	60,58	64,22	0,0392 *

**Table 4:** Mean values and results from the peak intensity comparison tests according to gender.

CONSISTENCY	AGE RANGES (YEARS)	MALES	FEMALES	p
	2 to 5	90,41	88,58	0,1477
Saliva	5 to 10	91,67	90,64	0,0631
	10 to 15	91,50	92,52	0,0434 *
	2 to 5	91,07	91,58	0,4412
Liquid	5 to 10	92,65	92,10	0,0559
	10 to15	92,50	92,62	0,2149
	2 to 5	90,08	90,00	0,9300
Paste	5 to 10	91,84	90,17	0,0277*
	10 to 15	91,46	92,42	0,0635

**Table 5:** Mean values and results from the swallowing time comparison test according to gender.

CONSISTENCY	AGE RANGES (YEARS)	MALES	FEMALES	p
	2 to 5	0,79	0,81	0,8223
Saliva	5 to 10	0,79	0,76	0,7520
	10 to 15	0,79	0,75	0,5396
	2 to 5	0,87	0,96	0,3088
Liquid	5 to 10	1,05	1,00	0,6274
	10 to15	0,99	0,97	0,7250
	2 to 5	0,81	0,83	0,7388
Paste	5 to 10	0,85	0,99	0,1096
	10 to 15	0,92	0,81	0,2605

### Significance in the same age range for saliva, liquid and pasty consistencies

The type of consistency interference in each one of the age ranges in the five variables studied is depicted by the p values for males (Table 6) and females (Table 7).

**Table 6:** Significance by variable in the three consistencies in the same age range (males)

AGE RANGE	FI	FP	II	IP	T
2 to 5 years	0,8741	0,5640	0,8743	0,5634	0,5807
5 to 10 years	0,1223	0,0380*	0,1225	0,0380*	0,0233*
10 to 15 years	0,9315	0,0689	0,9312	0,0679	0,1478

FI=initial frequency; FP=peak frequency; II=initial intensity; IP=peak intensity; T=swallowing time

**Table 7:** Significance by variable in the three consistencies for the same age range (females)

AGE RANGE	FI	FP	II	IP	T
2 to 5 years	0,0904	0,0085*	0,0901	0,0085*	0,1575
5 to 10 years	0,8539	0,0139*	0,8541	0,0139*	0,0104*
10 to 15 years	0,4127	0,6246	0,4125	0,6228	0,0014*

FI=initial frequency; FP=peak frequency; II=initial intensity; IP=peak intensity; T=swallowing time

Significance of the same consistency in the three age ranges

The p values for each one of the variables reflect the age interference in each one of the consistencies presented for males (Table 8) and females (Table 9).

**Table 8:** Significance for each consistency for the three ages studied (males).

CONSISTENCY	FI	FP	II	IP	T
Saliva	0,7283	0,2151	0,7283	0,2151	0,9970
Liquid	0,1171	0,0048*	0,1173	0,0048*	0,2140
Pasty	0,3312	0,0276*	0,3313	0,0276*	0,4439

FI=initial frequency; FP=peak frequency; II=initial intensity; IP=peak intensity; T=swallowing time

**Tabela 9:** Significancia para cada consistencia nas tres subfaixas etàrias (sexo feminino).

CONSISTENCY	FI	FP	II	IP	T
Saliva	0,0000*	0,0000*	0,0000*	0,0000*	0,6318
Liquid	0,0198*	0,0033*	0,0198*	0,0033*	0,6447
Pasty	0,0321*	0,0031*	0,0321*	0,0031*	0,0827

FI=initial frequency; FP=peak frequency; II=initial intensity; IP=peak intensity; T=swallowing time

## DISCUSSION

In a literature review we did not find any paper mentioning the use of this methodology in the age range between two and 15 years. There was only one paper involving Doppler sonar as a tool to capture and analyze swallowing sounds in adults, using different parameters from ours^1^.

By accepting that the larynx and oropharyngeal tract structures vibrate and move as they work as a system of valves and pumps causing the swallowing sounds,^23^ the many characteristics of these sounds would initially depend on the food consistency. However, they would also depend on gender and age as determining factors of the “resonance box” volume and energy applied to those structures based on modulators of those sounds.

The age range division, the use of different consistencies and the pairing among the individuals by gender aimed at trying to achieve a comparative analysis among the acoustic signal frequency and intensity variables and the sound wave time.

If we assume that during swallowing at a higher frequency and intensity a shorter time corresponds to a more efficient performance of the swallowing process, one could expect to have linear series and a scaled performance parallel to age progression and neurological maturity. Nonetheless, this did not happen to the majority of the modification series obtained.

Further studies, involving a larger number of participants may stress differences we did not find.

### Gender-related significance

As we consider initial frequency and intensity mean values associated with swallowing saliva, we notice a gender-related significance in the 2-5 and 10-15 year age ranges. In liquids and paste food, the significance is similar in the 5-10 (higher mean values among males) and 10-15 years (higher mean values among females).

The swallowing initial intensity and frequency mean values considering the three different food consistencies, in the age range of 10-15 years are always higher among females. Considering gender significance, this age range was the only one present in this variable among the three different food consistencies, which suggest better performance among girls (Table 1, Table 3).

As far as gender is concerned, the frequency and peak intensity variables were significant in saliva consistency from 10-15 years, and pasty consistency among those between 5 and 10 years of age.

The frequency and peak intensity mean values in all the three food consistencies studied are the lowest in the 2-5 years age range in comparison to the other ages, for both genders (Table 2, Table 4). When we consider that swallowing sounds involve laryngeal elevation^13^ we can notice that the lowest values found in these variables between 2-5 years in both genders would be caused by the lowest laryngeal dimension in that age range.

Frequency and peak intensity mean values are always higher in the 5-10 age range in males and in the 10-15 age range in females, in all the consistencies studied.

The high mean values in the 10-15 age range, in the peak and initial intensity and frequencies seem to point to a clear superiority in swallowing performance among females, with characteristics of an accelerated pace of development.

Considering liquid, the peak intensity and frequency mean values were always higher when compared by age range in relation to the other consistencies, both in males and females (Table 2, Table 4). The high values seen among these two variables for liquid swallowing suggest the need for higher upper airway protection with a higher laryngeal elevation in relation to swallowing saliva and pasty food.

There was no gender-related significance in the swallowing time variable in any of the age ranges or consistency type (Table 5).

In relation to saliva swallowing mean times, we noticed they were equal in all age ranges for males, showing uniformity as far as age is concerned. Nonetheless, among females there is a lowering in age ranges from younger to older ages, expressing an enhancement as time passes.

When we compared the swallowing time mean values among the three consistencies, considering the same sex and age range, saliva consistency swallowing requires a shorter time to be effective. When we analyze the mean values of this variable, we notice the inverse happening in relation to liquid consistency, where the highest values were found.

### Significance in the same age range in saliva, liquid and pasty consistencies

Among males, we noticed significance in the frequency and peak intensity variables and swallowing time only among those between 5 and 10 years (Table 6). Among females, we had significant values in the peak intensity and frequency in the age range from 2-5 years and 5-10 years of age. In relation to swallowing time, age ranges 5-10 and 10-15 years showed significance (Table 7).

### Significance for each food consistency in the three age ranges

The mean value variance within the same consistency, for the three age ranges, among males (Table 8) was seen for liquid and paste, only among frequency and peak intensity variables. Contrary to that, among females (Table 9), significance was marked for being present in all consistencies and in all the variables, except swallowing time.

## FINAL REMARKS

The data obtained from normal children may serve as comparison basis for our testing of pediatric patients with swallowing abnormalities, in order to establish disease patterns. On the other hand, the alterations seen in the tests of these children with anatomical and physiological alterations can serve for a better interpretation of the results hereby mentioned.

Swallowing sound interpretation and its correlation with physiological events which happen during this process do not seem yet clear. Being able to make this association will provide neck auscultation with greater credibility as instrumental diagnosis. The Doppler neck spectrography together with other swallowing assessment data such as videofluoroscopy and nasal-laryngeal fibroscopy can clarify the physiological causes of swallowing sounds.

## CONCLUSION

The Doppler sonar, gathering visual and acoustic data to detect swallowing sound characteristics, provided objective and measurable data. The variance analysis of the mean values found in each variable - frequency, swallowing time and intensity - proved significant in relation to gender, age range and food consistencies, with indications of a direct interference relation of swallowing and the biological development of the age range studied.
